# Citizen's Charter in a primary health‐care setting of Nepal: An accountability tool or a “mere wall poster”?

**DOI:** 10.1111/hex.12596

**Published:** 2017-07-21

**Authors:** Gagan Gurung, Robin Gauld, Philip C. Hill, Sarah Derrett

**Affiliations:** ^1^ Department of Preventive and Social Medicine Dunedin School of Medicine, University of Otago Dunedin New Zealand; ^2^ Otago Business School and Division of Commerce, University of Otago Dunedin New Zealand; ^3^ Centre for International Health, Department of Preventive and Social Medicine Dunedin School of Medicine, University of Otago Dunedin New Zealand; ^4^ Injury Prevention Unit, Department of Preventive and Social Medicine Dunedin School of Medicine, University of Otago Dunedin New Zealand

**Keywords:** accountability, citizen's charter, primary health care, transparency

## Abstract

**Background:**

Despite some empirical findings on the usefulness of citizen's charters on awareness of rights and services, there is a dearth of literature about charter implementation and impact on health service delivery in low‐income settings.

**Objective:**

To gauge the level of awareness of the Charter within Nepal's primary health‐care (PHC) system, perceived impact and factors affecting Charter implementation.

**Method:**

Using a case study design, a quantitative survey was administered to 400 participants from 22 of 39 PHC facilities in the Dang District to gauge awareness of the Charter. Additionally, qualitative interviews with 39 key informants were conducted to explore the perceived impact of the Charter and factors affecting its implementation.

**Results:**

Few service users (15%) were aware of the existence of the Charter. Among these, a greater proportion were literate, and there were also differences according to ethnicity and occupational group. The Charter was usually not properly displayed and had been implemented with no prior public consultation. It contained information that provided awareness of health facility services, particularly the more educated public, but had limited potential for increasing transparency and holding service providers accountable to citizens. Proper display, consultation with stakeholders, orientation or training and educational factors, follow‐up and monitoring, and provision of sanctions were all lacking, negatively influencing the implementation of the Charter.

**Conclusion:**

Poor implementation and low public awareness of the Charter limit its usefulness. Provision of sanctions and consultation with citizens in Charter development are needed to expand the scope of Charters from information brochures to tools for accountability.

## 1 BACKGROUND

An informed citizenry is a precondition for demanding accountability from service providers.^1^ Theoretically, citizen's charters are part of the New Public Management approach and are initiated to encourage service providers to be responsive and to inform citizens about service entitlements, standards and rights.^2^, ^3^ This approach envisages each citizen as a consumer and emphasizes individualism rather than collective notions of citizenship.^2^, ^3^

Citizen's charters were first implemented in the United Kingdom in 1991.^2^, ^4^ Soon after, the charter concept was adopted in various developed and developing countries.^5^ Although the central aim was to achieve better quality and a responsive service delivery, there are differences in intent, content and implementation in different countries.^6^ Despite the concept being variable, charters can be powerful accountability mechanisms, facilitating the expression of citizens’ expectations of service providers.

Nepal's primary health‐care (PHC) system includes networks of nearly 4000 peripheral health facilities.^7^ Health facilities include subhealth posts, health posts and PHC centres managed by district (public) health offices.^7^ These health facilities predominantly provide preventive and promotion services, with few curative services.^8^ A subhealth post is the first institutional contact point for basic health services.^8^ A community‐based service is provided by Female Community Health Volunteers (FCHVs) and outreach clinics managed by these health facilities.^8^ Subhealth posts act as referral centres for FCHVs and outreach services, which, respectively, refer on to health posts, to PHC centres and finally to hospitals.^8^ To ensure community voice in health facility management, health facility operation and management committees (HFMC) were formed in each these health facilities to manage funds, human resources and health programmes.^9^

In line with global trends, Nepal introduced the Citizen's Charter in 1998 as a reform initiative to modernize public service delivery.^10^ Nepal adopted a similar model to the United Kingdom. In the early 2000s, Nepal implemented Charters in PHC facilities.^1^ Citizen's Charters were uniform across all the health facilities.

Despite some empirical findings about the usefulness of charters with respect to awareness of rights and services in both developed and developing countries,^11^, ^12^, ^13^, ^14^, ^15^ there is a dearth of local literature about charter implementation and impact on health service delivery, particularly in rural settings. This article seeks to address this knowledge gap by reporting on awareness of the Charter, perceptions of the Charter's impact on transparency and accountability, and factors affecting the implementation of the Charter in a district in Nepal.

## 2 METHODS

### 2.1 Study setting

**Figure 1:**
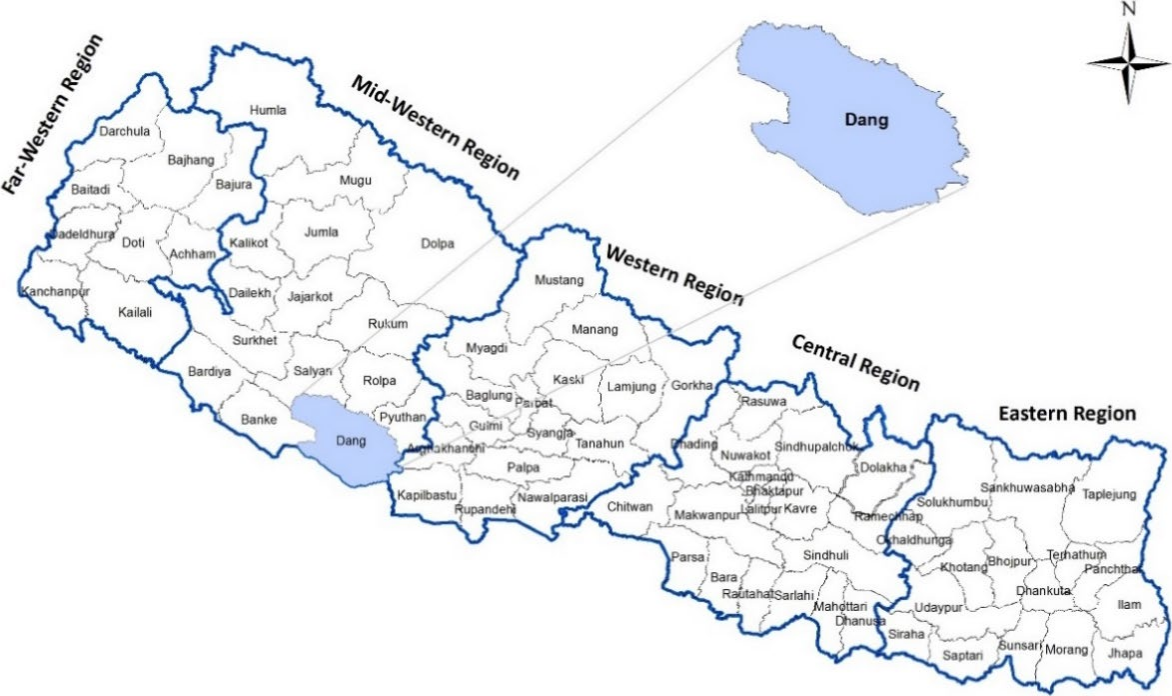
Map of Nepal showing the Dang District

This case study (2014‐2015) was conducted in Nepal's predominantly agricultural Dang District, located 280 km west of Kathmandu (Figure 1).^16^ Ecologically, the district contains both diverse topography and ethnicities. It has a population of approximately 550 000; nearly 80% live rurally.^16^ The literacy rate is 70%.^16^ The district health system of Dang contains networks of 39 PHC health facilities, including 21 subhealth posts, 15 health posts and three PHC centres, managed by the District Public Health Office.^7^

### 2.2 Data collection

This study was carried out as part of a larger project investigating social accountability mechanisms. Following pre‐testing, a structured face‐to‐face survey was administered by interviewers to 400 participants (220 service users, 100 HFMC members and 80 service providers) selected by two‐stage cluster sampling from 22 of 39 public health facilities in the Dang District. Selection of health facilities (clusters) at the first stage was used to reach the main sampling units (service providers/HFMC members/service users). Health facilities were stratified by subhealth posts, health posts and the PHC centres. Then, 22 health facilities (nine subhealth posts, 11 health posts and two PHC centres) were selected randomly from each strata. Service user flow of each sampled health facility was calculated, and the number of the service users to be interviewed in each health facility was determined in proportion to size. The required sample per health facility was selected systematically. Only five invited potential participants declined to participate. With respect to all potential HFMC members and service provider participants (n=219 and n=114, respectively), those available on the day of interview were approached and all those invited participated.

Questionnaires focused on gauging the awareness of health service providers and community people towards the Citizen's Charter. Awareness was assessed by knowledge of the existence of Charter in the health facility and understanding of the Charter's main features. Participants answered “yes” or “no” to a question asking whether they heard of the Charter in their health facility, and questions asked about the key features of the Charter which include types of services available, costs, time to receive services, complaints person, facilities and timetable of service hours. In addition, an audit was conducted at the 22 sampled facilities using a checklist to assess availability and visibility of the Charter.

The qualitative component of the study explored how Citizen's Charters were implemented, their perceived impacts and factors affecting the implementation of the Charter. Thirty‐nine interviews using open‐ended interviewing techniques were undertaken with HFMC members, service providers (health workers from the PHC facilities), district‐level health managers and non‐government organization (NGO) members. Six focus groups were also conducted with community people (one group with men, four with women and one with FCHVs). An interview guide included a list of topics and questions to be covered. Collecting data from diverse people with different backgrounds enabled triangulation of study findings.^17^

### 2.3 Data analysis

Pre‐coded quantitative data from the questionnaires were entered, checked for data quality and analysed using IBM SPSS Statistics 22.^18^ All the qualitative interviews and focus groups were audio‐taped, transcribed and analysed using NVivo 10.^19^ We used thematic analysis which included familiarization with the data by reading transcripts, open coding, development of coding framework and labelling data under the appropriate codes. The coded transcripts were summarized in narratives for each theme.

### 2.4 Ethics

We obtained ethical approval from the Human Ethics Committee of Otago University and the Nepal Health Research Council. Informed consent was obtained from all participants.

## 3 RESULTS

### 3.1 Socio‐demographics of participants

Among the 220 service users interviewed during the survey, 66% were female with a mean age of 33.7 years (range 19‐81 years) and more than two‐thirds (68%) used the health facilities for curative services. The majority (59%) were formally literate, and main occupations were those of housewife worker (41%) and agriculture worker (35%). In the case of service providers (n=80), most respondents were female (55%) and permanent workers (64%) with the mean duration of service of 11.5 years (SD 9.52; range=1‐38 years). In the case of HFMC (n=100), the majority of the members were male (62%) and literate (91%), with a mean duration of service of 4.3 years (SD 3.8; range=1‐20 years).

Of the 39 qualitative interviews, there were 34 male and five female participants with participation from community, health facility and district levels. Most of the participants were HFMC members (15), followed by service providers (10), NGO staff/members (10) and district‐level managers (4). Additionally, 56 participants (8 men; 48 women) made up the six focus groups; each focus group consisted of 8‐13 participants. There was one male focus group, whose members were ordinary citizens affiliated with different professions and also service users from the nearest public health facilities. Among the four female focus groups, most members were affiliated with community mothers’ groups or were service users. The one FCHV focus group participants represented different wards of the village development committees.

### 3.2 Knowledge of Citizen's Charter

The great majority of HFMC members (84%) and service providers (90%) had heard about the Charter. However, only 15% of service users reported awareness (Table 1). There was a tendency for men to be more aware of the Charter. A greater proportion of service users who were literate had heard of the Charter compared to those who were illiterate, and there were also some differences according to ethnicity and occupational group. Of the service user respondents who had heard of the Charter (n=33), two‐thirds (n=22) had actually read it.

**Table 1:** Characteristics of service users in relation to knowledge of the Citizen's Charter (n=220)

Service user characteristics	Heard of Citizen's Charter		χ^2^	*df*	*P*‐value
	Yes	No			
	n (%)^a^	n (%)^a^			
All (n, %)	33 (15)	187 (85)			
Sex					
Male	16 (21)	60 (79)	3.336	1	.07
Female	17 (12)	127 (88)			
Literacy					
Illiterate	1 (2)	47 (98)	8.034	1	.005
Literate	32 (19)	140 (81)			
Ethnicity					
Dalits (marginalized caste)	4 (11)	32 (89)	10.337^b^	3	.02
Adhivasi/Janajati (indigenous/ethnic group)	7 (8)	85 (92)			
Terai caste	4 (29)	10 (71)			
Brahman/Chhetri (upper caste)	18 (23)	60 (77)			
Occupation					
Housewife	13 (15)	76 (85)	22.173^b^	3	.000
Agriculture	5 (7)	71 (93)			
Fulltime employed	15 (38)	25 (63)			
Casually employed	0 (0)	15 (100)			

a Percentage may not equal 100 due to rounding.

b Likelihood ratio.

The interviews and focus groups also revealed that the majority of the citizens were not aware of the existence of the Charter in their health facilities:

Few people may know about it. We do not know about it. You told us today for the first time, thus we will check it next time when we go to the hospital [PHC centre] for health service. Generally, we are only concerned with our problems and do not pay attention to other things. (Focus group, community people‐female group in PHC centre 2)

### 3.3 Knowledge of participants on the main features of the Citizen's Charter

**Figure 2:**
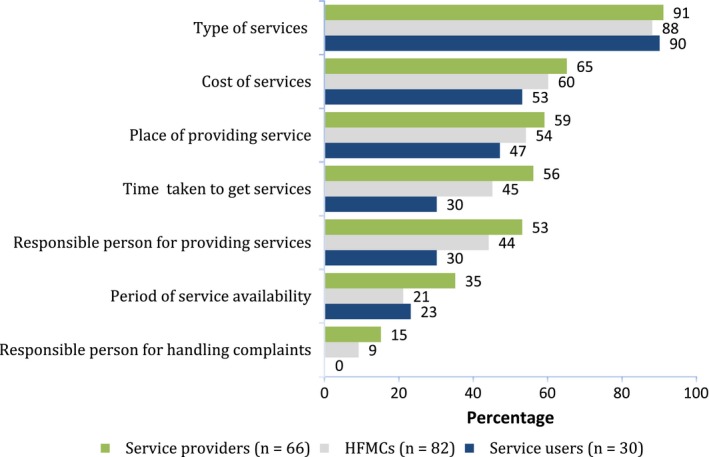
Respondent's knowledge of the main features of the Charter (%)

There is a guideline about the minimum information to be incorporated in a Nepalese Citizen's Charter.^20^ Based on this guideline, a separate question was asked of respondents who reported awareness of the Charter about their understanding on each of these elements/aspects (Figure 2). Among 33 service users, 84 HFMC members and 72 service providers who reported the awareness of the Charter, only 30, 82 and 66 participants, respectively, responded to this question. Types of services available and the cost of services were the most frequently cited features of the Charter, and a person responsible for handling complaints was the least‐mentioned feature of the Charter among all three groups.

### 3.4 Source of knowledge about the Charter among service users (n=33)

**Figure 3:**
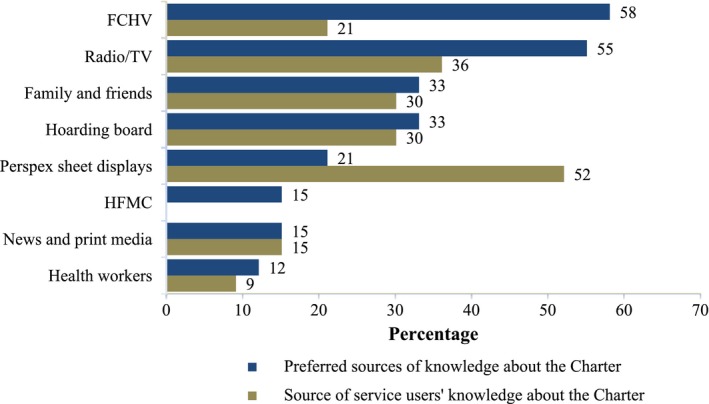
Source of knowledge about the Charter among service users (%)

Respondents knew mostly about the Charter through displayed perspex sheets (52%) followed by radio/television information (36%) (Figure 3). However, they felt that FCHVs (58%) and radio/television (55%) would be the preferred means of learning about the Charter. Although they had not heard about the Charter from HFMC, 15% of the respondents perceived that the HFMCs would also be a useful way of being informed about the Charter.

### 3.5 Perceived impact of Citizen's Charter

In the survey, service users were asked about the overall usefulness of the Charter in terms of informing them about health facility services. Qualitative components explored in more detail the usefulness of the Charter in holding service providers accountable and improving health service delivery.

#### 3.5.1 Usefulness of the Charter in learning about the service

The survey showed that of the service user respondents who had heard of the Charter (n=33), 20 (61%) believed the Charter was readable and understandable for only a small proportion of the general public. However, a good majority of service users (67%) felt that the Charter was helpful to some extent in getting information about the service they wanted from the health facility.

#### 3.5.2 Role of Charter in transparency

Interviews and focus groups showed that the Charter was seen as a potential tool to increase transparency, particularly for those who were educated and had a habit of reading information. However, the Charter was not useful for illiterate people:

When service users come and read the Charter, they know about the service available from a health post and can behave accordingly. I mean it makes clear to service users what the service is that they can get from health post and what service providers provide, thus they can ask for services they needed. It is like a mirror. It mirrors services available from the health post and it encourages them [users] to utilise services. However, it is only useful to those who can read it. (Qualitative interview, HFMC member 1, health post 3)

Some thought the Charter had not led to increased transparency and was not able to make the mandate of the health facility services clear to citizens. Indeed, the general public were largely unaware of the facility and its services, limiting the role the Charter could play.

People come and make complaints about why we do not get X‐ray services or blood examinations in the sub‐health post. When I referred patients in the district [hospital] to have a lab test, they questioned ‘why you do not provide the service here? It should be provided here’. The problem is they do not know what level of staff are available at the sub‐health post. So this confusion is due to the lack of awareness of the general public on the standard and level of services available here which should have been made clear by the Charter. (Qualitative interview, clinic manager, subhealth post 2)

We do not know about it [the Charter]. You told us today for first time, thus we will check it the next time when we go to the hospital [PHCC]. Generally, we are only concerned about our health problems and do not give attention to other things. (Focus group, community people—female group in PHC centre 2)

It appeared that the Charter's role in transparency was limited to being on display as an information board. Regarding information disclosure in health facilities, an important part of transparency, many health facilities, displayed a list of the drugs and the organizational chart of the facility, and a list of HFMC members and FCHVs. However, the drug lists were in English and many citizens could not read it. Furthermore, the information was not displayed in an appropriate place. A few health facilities also displayed the monthly update of mothers who received a maternity incentive under the Government's free delivery policy (“Aama”). However, this was also poorly displayed. Similarly, some health facilities displayed the annual and monthly programme targets and achievements. None of the health facilities displayed their financial information. Furthermore, qualitative interviews revealed that information disclosure in a health facility could not be attributed to the Charter. The disclosure was reported as “business as usual” and not as a consequence of the Charter.

#### 3.5.3 Role of Charter in raising citizen's concerns

It is plausible to think that citizens can complain if a service is not provided in accordance with the standard mentioned in the Charter. However, such an assumption did not hold true for the Charter in this study. One of the health post managers commented, “*I have been serving here for five years, but no one has ever asked me about why service in the Charter is not available in the health post” (Qualitative interview, clinic manager, subhealth post 2)*.

#### 3.5.4 Role of the Charter in improving service providers’ accountability

Interviewees generally perceived that Charters were found to be of limited use in holding service providers accountable:

It may have an impact if staff follow it, but in our case it has no effect. Our health post opens only until 2 o’ clock. Staff do not follow what is written in the Charter. So just imagine how the Charter is implemented. There is a discrepancy between what is in the Charter and what is actually implemented. (Qualitative interview, Dalit (marginalized caste) HFMC member, health post 1)

By rules, one should abide by the Charter. However, you know some [staff] come at 12 and go at 2 o'clock. Where is [the impact of] the Charter? It is just like other posters mounted on the wall. (Qualitative interview, auxiliary nurse midwife, health post 2)

However, a few participants felt that Charters had some effect on staff accountability and health service improvement by making service provision “favourable and accessible.” One HFMC member mentioned, “It has some effect on making health workers alert” (qualitative interview, HFMC member 2, health post 3). Similarly, a HFMC chairperson mentioned, “[Due to the Charter] the public become aware of what their rights are and the services available from the health facility. For health workers, it reminds them about their responsibilities” (Qualitative interview, chairperson, PHC centre 1).

A District Public Health Office participant also had a positive view towards the perceived impact of the Charter.

At least the Charter has provided a guideline about health services. I think in our district, we are able to meet 75% of the standards [mentioned in the Charter] by providing basic services from the health facilities. But to meet all the standards, there is a need for improvement in wider areas and resources. (Qualitative interview, high‐level health planner, District Public Health Office)

### 3.6 Factors affecting the implementation of the Charter

Eight different factors emerged from the qualitative component as affecting the implementation of the Charter: lack of proper display, lack of consultation with stakeholders, lack of orientation or training, no follow‐up and monitoring, lack of enforceability, service providers’ attitude and illiterate citizens.

#### 3.6.1 Lack of proper display

The health facility audit showed that the Charter was found in 19 (86%) of 22 observed health facilities with 12 (84%) of the Charters containing up to date information. However, only three (16%) Charters were in a visible location within the clinics. Qualitative interviews and focus groups also found that in many places, the Charter was not adequately displayed or simply put in the cupboard and that the displayed information was incomplete. In addition, there was no monitoring of the quality of implementation:

If it is displayed in a visible place at least educated people may read it. If it is dumped somewhere in the room then how can people know anything from it? (Focus group, community people—female group in health post 1)

The reasons reported for not displaying the Charter outside the health facilities included the following: information secrecy by health workers and fear of it being stolen or getting damaged.

#### 3.6.2 Lack of consultation with stakeholders

The implementation process was top‐down. Consultation with citizens while designing and implementing the Charter was non‐existent. Hence, very few citizens knew about the existence of the Charter.

There was no consultation with the public, nor with HFMC/staff either. It was an exported thing because someone gave it to us and we hung it on the wall. If the Charter was prepared by the public consultation, then it would have been like a mirror, but if it was just hung on the wall which was handed over by someone then it became a mere wall poster. (Qualitative interview, HFMC member 2, health post 3)

#### 3.6.3 Lack of orientation or training

There was no orientation or training provided to either health service providers or citizens regarding the Citizen's Charter. Hence, many respondents understood it as a mere information tool just like other posters hung on the wall, but not as an accountability tool.

There is no orientation for us about why the right to information is necessary or why Charter is needed. There is only [direction from the District Public Health Office] that we should have one displayed, and there is no specific budget allocated for it. What the District Public Health Office did was they sent ready‐made perspex and what we did was just to mount it on the wall. There was no specific training about the Charter. (Qualitative interview, clinic manager, PHC centre 2)

#### 3.6.4 No follow‐up and monitoring

The practice of follow‐up and monitoring of the implementation of Charter was almost non‐existent. In addition, the indicators related to Charter were not included in the integrated supervision and monitoring checklist of the district health system:

There is never a discussion about, or monitoring of, the Charter. There is no discussion on whether it is displayed properly or what its effect is. (Qualitative interview, clinic manager, PHC centre 2)

#### 3.6.5 Lack of enforceability

Qualitative interviews revealed that the Charter has no role in enforcing any positive or negative sanctions. There was no provision in the Charter for appropriate action if health services were not held to the standards mentioned in the Charter.

The Charter is just like a toothless tiger. […] no effect on staff accountability. For example, it is written in the Charter that the opening hours of the health post are from 10 am to 5 pm, but in fact, it closes before 2 pm. There is no provision [in the Charter] to take action if services mentioned in it are not provided. What is necessary is to add [to the Charter] steps to be taken if service is not delivered according to the Charter. (Qualitative interview, HFMC member, health post 3)

#### 3.6.6 Service providers’ attitude

It was found that service providers’ attitude and information secrecy was another reason for poor implementation of the Charter. For example, service providers gave little emphasis to the Charter as they did not feel responsible for providing services according to the Charter, and the implementation of the Charter was top‐down. In some cases, they were found to intentionally not display the Charter in a visible place promoting information secrecy:

The main problem is the mentality of the health workers that they are not ready to take responsibility and to provide the services as mentioned in the Charter. What I think is that health workers have given little importance to it. For example, the Charter is placed in the health facilities [by health workers] not with the intention of letting public know about the services and providing them services guided by the Charter, but just to show the officials coming for supervision that they have followed the government's guideline. (Qualitative interview, district supervisor 3, District Public Health Office)

The Citizen's Charter gives details about the service given by health facilities. It is clearly written that the health facility is to be opened from 10 am to 5 pm. But our staff stay from 10 am to 1 pm only. Informed people might ask questions, so that may be one reason why it [the Charter] is put inside [a cupboard] folded [away]. (Qualitative interview, HFMC member 1, health post 1)

#### 3.6.7 Illiterate citizens

Illiterate people cannot read the contents of the Charter, restricting its usefulness to educated people only:

Those [service users] who are educated they might look into it [the Charter]. But for those who are uneducated and marginalized, it has no meaning. The volume of such service users is high. (Qualitative interview, clinic manager, subhealth post 1)

Related to above, there was lack of a practice among citizens in reading and taking interest in health matters which also greatly affected the Charter's usefulness:

Generally, the public are concerned about their own health problems or services they want to get. So very few people give attention to the Charter. (Qualitative interview, auxiliary nurse midwife, health post 2)

## 4 DISCUSSION

The main aim of this study was to understand the level of awareness of the Charter, the perceived impact and factors affecting the implementation of the Charter in a rural and remote PHC setting, using the Dang District as the case study. This appears to be the first comprehensive study of various aspects of a Citizen's Charter in a primary care context in a low‐income setting.

This study found a low level of awareness of the Citizen's Charter among citizens which was consistent with a previous study in Ghana and Kenya.^21^, ^22^ If the majority of service users were unaware of the existence of the Citizen's Charter in the health facilities, it is not hard to appreciate that the Charter is not an effective transparency and accountability tool, and this raises questions regarding its design and implementation.

Regarding the perceived impact of the Charter, its role in enhancing transparency and accountability was found to be limited. From the accountability point of view, a key contribution of a charter is to make the standards and mandate of the health facility service clear^23^ so that citizens know what to expect from the PHC system. However, this study showed that Charter had not led to increased transparency and was useful for only few educated citizens. Another key assumption about a charter is that it informs citizens about their rights so they can, in turn, exert voice or make complaints about service providers to improve performance^1^ which is also found to be poor in the present study. Most importantly, in contrast to findings from a health facility charter in Kenya,^15^ the Charter's role in improving health service provider accountability and health service improvement was not strong in Nepal.

Many factors emerged as reasons for the poor implementation of the Charter including: lack of proper display of the Charter, lack of prior consultation with stakeholders, lack of orientation and training of stakeholders, lack of follow‐up and monitoring and lack of provision of sanctions in response to the Charter. Factors related to service providers was information secrecy. Illiteracy and lack of established culture were community‐related factors. These findings echo results in other studies conducted in Ghana^22^ and Kenya.^15^

While a citizen's charter is intended to be an accountability tool oriented to rights of the individual rather than at the collective notion of citizens,^2^, ^3^ in a health‐care context in a developing country, it appeared that use of such individual accountability mechanisms may not be entirely appropriate. In contexts where many citizens are illiterate and not empowered and there is no strong tradition of consumer rights,^2^ mechanisms which emphasize an organized participation of citizens for collective goals may be more appropriate to enhance service providers’ accountability.^24^, ^25^

In contrast with other studies on citizen's charters, which were mostly from outside the health service delivery contexts,^5^, ^11^, ^12^, ^13^ this study adds new insights by exploring the level of awareness and perceived impact of the Charter and by identifying the design and implementation challenges of the Charter in a health system context. As the charter concept is adapted from developed countries, this study highlighted the relevance and challenges of implementing the concept in a rural and underdeveloped service delivery context. Hence, these findings provide a useful evidence base to strengthen the charter concept in health service delivery in rural and low‐income settings. A number of policy and practical implications arise from the findings. These include the following.

### 4.1 Consultation with stakeholders

Consultation with citizens and front‐line service providers while designing and development of the Charter is necessary. Such process would give an opportunity to design the Charter based on local needs, build trust and support for its implementation and raise awareness of the Charter.

### 4.2 Provision of sanctions in the Charter

There needs to be provision within the Charter to take appropriate action if health services are not according to the standards mentioned in the Charter. A functioning grievance redress mechanism should be established.^2^ Such provision within the Charter would likely help improve service provider accountability and service improvement.

### 4.3 Awareness of the Charter

An important area, highlighted by this research, is the need to increase the level of awareness of citizens with respect to the Citizen's Charter. The Citizen's Charter should be displayed properly, in a visible place, with all the necessary information about the service delivery standards. Since awareness is low among women and those with limited literacy, a particular focus is needed for raising awareness among these groups. Also, since it appears that the Charter is useful only for those who can read it, an alternative approach to publicizing the Charter is recommended. This study shows that many preferred to know about the Charter through FCHVs or radio/television. Nepal's Demographic Health Survey (2011) showed that more than half Nepalese households have access to radio/television, and three‐quarters have a mobile telephone.^26^ Such channels could be promoted to increase awareness about the concept and contents of the Charter in the rural community setting of Nepal, instead of simply relying on written materials.

### 4.4 Orientation and training to service providers and HFMC members

The concept of the Charter was not properly understood, and there was a need to change the mindset of service providers. Orientation and sensitization to service providers and committee members seemed important to make them aware about the concept and spirit of the Charter.

### 4.5 Monitoring and follow‐up of the Charter

The Charter initiative remained a one‐shot activity with lack of follow‐up and monitoring of how it is implemented or its effectiveness. There is a need to develop a mechanism to monitor its implementation by integrating activities related to the Charter in existing supervision and monitoring checklists of the district health system.

### 4.6 Strengths and limitation of the study

Strengths of the study include that data were collected from participants with different backgrounds representing different levels of health facilities and varied geographies which helped to triangulate the findings. Furthermore, the lead author himself was involved in conducting all the qualitative interviews which helped ensure a deep understanding of the phenomena. However, this study does have limitations. There may be a number of response biases while conducting a questionnaire survey due it being based on personal opinion. However, pre‐testing of the questionnaires and training of research assistants (who were locals) helped to reduce the biases. Although we wanted to include the perspectives (voices) of service users and the general public in the qualitative component of the study, due to the Charter concept being relatively new and complex, we recruited more service providers and HFMC members who were thought to be “information‐rich” sources.^27^ Furthermore, we included the perspectives of service users/general public in the focus groups and survey interviews. It was not possible in this cross‐sectional study to measure the impact of the Charter against health service delivery improvements. With the maturity of the Charter implementation process, a future study could inform in the impact of the Charter programme in different aspects of health service delivery.

## 5 CONCLUSION

Poor design, development and implementation, and low public awareness of the Charter have limited the function of the Charter a mere information tool. Unlike its relevance in developed countries, one wonders whether the Charter concept is really an effective transparency and accountability tool in the health‐care context of developing countries like Nepal where a significant portion of the citizens are illiterate,^28^ where the culture of seeking information by reading information is not well established, and where consumer rights have not been well explained. That said, provided adequate attention is given to design and development of the Charter, to implementation and monitoring and to linking the Charter to health service improvement, it seems there is potential to realize the Charter's values and principles.

## CONFLICTS OF INTERESTS

The authors declare that they have no competing interests.
